# PiRNA-63049 inhibits bone formation through Wnt/β-catenin signaling pathway

**DOI:** 10.7150/ijbs.64533

**Published:** 2021-10-25

**Authors:** Gaoyang Chen, Shang Wang, Canling Long, Zhenmin Wang, Xin Chen, Wanze Tang, Xiaoqin He, Zhiteng Bao, Baoyu Tan, William W Lu, Zhizhong Li, Dazhi Yang, Guozhi Xiao, Songlin Peng

**Affiliations:** 1 Department of Spine Surgery, the Second Clinical Medical College, Jinan University (Shenzhen People's Hospital), Shenzhen Key Laboratory of Musculoskeletal Tissue Reconstruction and Function Restoration, Shenzhen 518020, China.; 2 The First Affiliated Hospital, Jinan University, Guangzhou 510630, China.; 3 The First Affiliated Hospital, Southern University of Science and Technology, Shenzhen 518055, China.; 4 School of Medicine, Southern University of Science and Technology, Guangdong, Provincial Key Laboratory of Cell Microenvironment and Disease Research, Shenzhen Key Laboratory of Cell Microenvironment, Shenzhen 518055, China.; 5 Department of Orthopaedic and Traumatology, The University of Hong Kong, Hong Kong, 999077 China.

**Keywords:** Bone formation, Bone marrow stromal cells, Piwi-interacting RNAs, Wnt signaling

## Abstract

Bone remodeling is a dynamic process between bone formation mediated by osteoblasts and bone resorption mediated by osteoclasts. Disrupted bone remodeling is a key factor in postmenopausal osteoporosis, a metabolic disorder characterized by deteriorated bone microarchitecture and increased risk of fracture. Recent studies have shown that piwi-binding RNA (piRNA) is involved in the pathogenesis of certain diseases at the post-transcriptional level. Here, we analyzed piRNA-63049 (piR-63049), which may play an essential role in bone remodeling. The expression of piR-63049 significantly increased in both bone tissues and plasma of osteoporotic rats and postmenopausal osteoporotic patients. Overexpressing piR-63049 could inhibit the osteoblastogenesis of bone marrow stromal cells (BMSCs) while knocking down piR-63049 could promote the osteoblastogenesis of BMSCs through the Wnt2b/β-catenin signaling pathway. Moreover, knocking-down piR-63049 (piR-63049-antagonist) *in vivo* could attenuate the bone loss in ovariectomized rats by promoting bone formation. Taken together, the current study shows that piR-63049 inhibits bone formation through* the* Wnt2b/β-catenin signaling pathway. This novel piRNA may be a potential target to increase bone formation in bone loss disorders such as postmenopausal osteoporosis.

## Introduction

Postmenopausal osteoporosis is a metabolic disorder characterized by bone loss and deterioration of bone microstructure, which ultimately leads to an increased risk of fracture 1-3. The risk of fracture in postmenopausal women is as high as 40%, which mainly occurs in the spine, hip, and distal radius 4, 5. Osteoporotic fractures and related complications lead to increased morbidity and mortality in the elderly 5, 6. The homeostasis of bone is maintained by bone remodeling, a dynamic process between osteoblast-mediated bone formation and osteoclast-mediated bone resorption 7. Disrupted bone remodeling is characterized by a decreased capacity for bone formation and increased bone resorption due to loss of estrogen, which is the leading cause of postmenopausal osteoporosis 8, 9. Bone marrow stromal cells (BMSCs) are the precursor cells of osteoblasts, which have been found to have decreased capability of osteogenic differentiation and bone formation in postmenopausal osteoporosis patients 10, 11. However, the underlying mechanism of decreased bone formation capacity of BMSCs from osteoporotic patients is still not fully elucidated. Therefore, an in-depth understanding of bone metabolism, especially the osteoblastogenesis of BMSCs, will help illuminate the biology of disrupted bone remodeling in osteoporosis as well as identify novel targets for the prevention and treatment of postmenopausal osteoporosis.

Noncoding RNAs (ncRNAs) have been found to be important in bone remodeling and bone metabolism in the last decade 12. Some studies have identified the regulatory roles of micro RNAs (miRNAs), long noncoding RNAs (lncRNAs), and circular RNAs (circRNAs) in osteoblastogenesis of BMSCs 13-16. Piwi-interacting RNAs (piRNAs), a newly discovered subclass of small ncRNAs, perform their regulatory functions by explicitly interacting with Piwi proteins 17-19. PiRNAs were first ncRNAs identified to be germline-specific, and play critical roles in the differentiation and maintenance of germ cells 20, 21. Recently, multiple pieces of evidence indicated that piRNAs were expressed in somatic cells, some of which played pivotal roles in eliminating mRNAs at the post-transcriptional level thereby affecting disease progression 22-28. PiRNA bears a 2'-O-methyl-modified 3' termini which enables it to interact with PIWI proteins 29. Piwi can guide a piRNA to recognize and silence the target mRNA 30. Furthermore, recent studies have found piRNAs were expressed in the exosomes of BMSCs, and they were also dynamically expressed during the osteogenic differentiation process of BMSCs, indicating piRNAs might play a critical role in osteogenesis 31, 32. However, whether piRNAs have a regulatory function in bone remodeling in postmenopausal osteoporosis was unknown.

To explore the role of piRNAs in bone remodeling and bone loss disorders, we carried out RNA sequencing of BMSCs from ovariectomized (OVX) rats and identified piR-63049 as an osteoporosis-related piRNA. We further established that piRNA-63049 plays a pivotal role in controlling bone mass in the rat. Mechanistically, using a combination of* in vitro* and* in vivo* strategies, we demonstrate that piR-63049 negatively regulates bone mass by targeting the Wnt2b in BMSCs. To our knowledge, this is the first demonstration of piRNA in the regulation of bone formation.

## Materials and methods

### Animal Experiments

The Sprague Dawley rats used in the present study were purchased from the Medical Experimental Animal Center of Guangdong Province. All experiments involving rats were approved by the Animal Ethics Committee of Shenzhen People's Hospital. Eight-week-old female rats were anesthetized and randomly divided into two groups, which then underwent either bilateral removal of ovaries (ovariectomized operation) or sham operation 33. Penicillin was injected intramuscularly at a dose of 80,000 units per rat for three days. Two months later, the bone microstructures of the OVX rats were evaluated by micro-CT scan. The study was approved by the research ethics committee of Shenzhen People's Hospital (NO. LL-KY-2020154).

### BMSCs isolation and culture

The bone marrow from OVX and sham rats was collected and the BMSCs were separated by density gradient centrifugation and then cultured in Alpha Modified Eagle's Medium (Gibco, Thermo Scientific, CA, United States) with 10% fetal bovine serum (FBS, Gibco, Thermo Scientific, CA, United States) and 1% penicillin and streptomycin 34. Flow cytometry was used to detect cell surface antigens, with BMSCs identified as negative for CD34 and CD45 while positive for CD29 and CD90 35.

Human BMSCs were purchased from American Type Culture Collection (ATCC, Manassas, United States) and cultured in Alpha Modified Eagle's Medium with 10% fetal bovine serum and 1% penicillin and streptomycin. All cells were cultured in an incubator with 5% CO_2_ and 95% humidity.

### Human plasma and bone tissue

Postmenopausal women (aged between 60 and 70 years old) subjected to spinal fusion surgery due to spinal canal stenosis in Shenzhen People's Hospital were included in this study. Those patients diagnosed with osteoporosis according to bone mineral density measurement (T score ≤ -2.5 SD) were included in the experimental group while those without osteoporosis (T score ≥ -1.0 SD) were in the control group (Supplementary Table 1). Additionally, patients diagnosed with other metabolic diseases (such as diabetes, rheumatoid arthritis, and osteonecrosis) were excluded from this study 33. A written informed consent was received from each participant prior to inclusion in the study. Peripheral blood was collected from all patients before surgery, and plasma was separated by density gradient centrifugation and stored at -80 °C. Approximately 100 mg of bone tissue was collected from each patient during the operation and then stored at -80 °C.

### RNA sequencing and identification of dysregulated piRNAs

Total RNA of BMSCs from three OVX and three sham rats was extracted using the Trizol method (Invitrogen, CA, United States). RNA sequencing was then performed by RiboBio (RiboBio Co. LTD, Guangzhou, China) to screen the expression profile of piRNA in BMSCs. In brief, small RNA was reverse-transcribed to cDNA. The raw data was generated by high-throughput sequencing using Illumina HiSeq^TM^ 2500. The sequencing data was then compared with the bioinformatics database of piRNA, and the information of piRNA expression in the database was obtained, including structure, length, expression level. The clean data obtained by the sequencing was compared with all piRNA sequences in piRBase database by blast software and its expression level was calculated. Any piRNA with a fold change ≥ 2 and p < 0.05 were considered as differentially expressed piRNA 34. Heatmaps and volcano plots were then constructed to show the expression of these dysregulated piRNA. Heatmaps and volcano plots were then constructed to show the expression of these dysregulated piRNAs and mRNAs.

### Quantitative real-time PCR

Total RNA of plasma, bone tissues and BMSCs was extracted using AG RNAex Pro Reagent (AG21102, Accurate Biotechnology, Hunan, China). The cDNA used for piRNA quantitative analysis was reverse transcribed from total RNA using the piRNA First Strand cDNA Synthesis kit (Tailing Reaction, Sangon Biotech Co. Ltd., Shanghai, China). The cDNA used for mRNA quantitative analysis was reverse transcribed from total RNA using the PrimeScript ^RT^ reagent Kit (RR037A, Takara, Tokyo, Japan). All the primers used in this study were designed and synthesized by Sangon Biotech Co. Ltd. (Shanghai, China) (Supplementary Table 2). Quantitative real-time PCR (qPCR) was performed using TB Green Premix Ex Taq (RR420A, TAKARA, Tokyo, Japan) on a StepOne Plus quantitative PCR system (Applied Biosystems, Foster City, CA, United States). The relative expression of piRNAs and mRNAs were calculated using U6 and glyceraldehyde-3-phosphate dehydrogenase (*Gapdh*) as internal reference standard genes, respectively, using the 2^-ΔΔCt^ method.

### PiRNA-mRNA network

PiRNAs can exhibit a miRNA-like mechanism in the cytoplasm, that is, by binding to the 3 '- UTR of target mRNAs and then inhibiting the expression of these target mRNAs. Therefore, we constructed a piRNA-mRNA regulatory network by combining miRanda, Pita (combined with our local database), and Rnahybrid databases and mRNA differential expression profiles with Cytoscape software.

### Dual-luciferase reporter assay

The luciferase plasmids containing wildtype or mutant *Wnt2b* (3 '- UTR sequence) were designed and synthesized by OBiO Technology Co. Ltd. (Shanghai, China) (Supplementary figure 1). 293T cells were seeded into 24 well plates at a density of 2 × 10^5^ cells per well and cultured in an incubator with 5% CO_2_ and 95% humidity. The 293T cells were transfected by above luciferase plasmids with or without piR-63049 mimics by GoldenTran-DR (Golden Trans Technology, Changchun, China). Renilla luciferase was used as the internal control. After 48 hours of transfection, the firefly luciferase and renilla luciferase activity were separately measured using a fluorescence spectrophotometer (Infinite M200, Tecan, Switzerland) following the manufacturer's instructions for the Dual-Luciferase Reporter Assay kit (Promega, USA). The relative luciferase activities were analyzed by normalizing firefly luciferase activity with renilla luciferase activity. Finally, the results showed the relative luciferase activity was significantly decreased in 293T cells co-transfected with a variant 3'-UTR sequence of Wnt2b and piR-63049 mimics.

### Western blot

Total proteins of BMSCs or bone tissues were extracted using the Total Protein Extraction Kit (Signalway Antibody LLC, Maryland, USA) according to the manufacturer's instructions. SDS-PAGE gels were utilized to separate proteins, which were then transferred onto PVDF membranes. Next, the PVDF membranes were incubated with the primary antibodies against β-catenin (Santa Cruz Biotechnology, USA; sc-7963; 1:1000), Wnt2b (Abcam, UK; ab178418; 1:1000), ALP (1:1000, Santa Cruz Biotechnology, USA), Runx2 (Cell Signaling Technology, USA; 12556; 1:1000), Ocn ( Santa Cruz Biotechnology, USA; sc-390877; 1:1000), Opn (Santa Cruz Biotechnology, USA; sc-21742; 1:1000), Lamin B1 (Abcam, UK; ab16048; 1:5000), and, Gapdh (Abcam, UK; ab9485; 1:5000) overnight at 4 °C, after which the corresponding secondary antibodies were applied for 1 h at room temperature. Finally, RapidStep™ ECL Reagent (Millipore, Germany) was used to visualize the protein bands, with GAPDH used as the loading control. Finally, RapidStep™ ECL Reagent (Millipore, Merck KGaA, Darmstadt, Germany) was used to visualize the protein bands.

### Immunofluorescence staining of BMSCs

BMSCs were seeded into 24 well plates with slides and incubated at 37 °C with 5% CO_2_ and saturated humidity. 48 hours post-transfection, the cells were fixed in ice-cold 4% paraformaldehyde for 30 min. Permeabilization was performed with 0.25% Triton X-100 in PBS for 10 min followed by blocking for 1h with 10% Goat serum. The fixed cells were then incubated with 1:100 dilution of PIWI (Abcam, UK), β-catenin (Santa Cruz Biotechnology, USA), and Wnt2b (Santa Cruz Biotechnology, USA) overnight at 4 °C. After washing three times with PBS, the slides were incubated with fluorescein isothiocyanate (cy3)-conjugated secondary antibody in a 1:200 dilution in PBS for 1 h at room temperature in darkness. The nuclei were subsequently stained with 4,6-diamidino-phenyindole (DAPI) (Abcam, Cambridge, UK) and the slides were then examined using Leica DMi8 microscope (Carl Zeiss MicroImaging GHBH; Jena, Germany). Statistical fluorescence intensity was performed by using ImageJ as the previous study described 36.

### Overexpression and knockdown of piR-63049 and *Wnt2b*

The inhibitor (sequence: GGAGACACGUGCACUGUAGA) and mimics (sequence: UCUACAGUGCACGUGUCUCC) of piR-63049 were designed and synthesized by Genepharma Biotech Co. Ltd. (Suzhou, China) to prevent and increase piR-63049 function, respectively. The small interfering RNA (si-*Wnt2b,* sequence: GCCAAAGAGAAGAGGCUUATT, UAAGCCUCUUCUCUUUGGCTT) and overexpression plasmid of *Wnt2b* (ov-*Wnt2b*) were designed and synthesized by OBiO Technology Co. Ltd. The above RNAs and plasmids were transfected into BMSCs with Lipofectamine™ 2000 Transfection Reagent.

### Osteogenic induction

The human BMSCs were cultured with MSC Osteogenic Differentiation Basal Medium supplemented with 10% FBS, 10 mM β-glycerophosphate, 0.1 μmol/L dexamethasone, and 50 μg/mL ascorbate (Cyagen Biosciences, Guangzhou, China) and incubated at 37 °C with 5% CO_2_ and saturated humidity. The osteogenic medium was changed every two days.

### Alizarin red and ALP staining

To determine the osteogenic differentiation of BMSCs transfected with si-*Wnt2b* or ov-*Wnt2b*, the cells were seeded in 48-well plates and incubated at 37 °C with 5% CO_2_ and saturated humidity. The BMSCs were stained at days 3, 7, and 10 after transfection and osteogenic induction. The calcium deposit was stained with 1% alizarin red staining kit and pictures were taken by microscopy (TS2-S-SM, Nikon, Japan). The osteogenic differentiation of BMSCs transfected with piR-63049 mimics or inhibitor was detected by ALP stain using the BCIP/NBT Alkaline Phosphatase Color Development Kit (Beyotime Biotechnology, China) at day 7 after transfection and osteogenic induction, according to the protocol. Then the cells were imaged using a Canon EOS 350D digital camera, placed vertically in a stable manner over a white background. Alkaline Phosphatase Assay Kit (Beyotime Biotechnology, China) was used to detect the ALP activity and the manufacturer's instruction.

### AntagopiR-63049 injection

The piR-63049-antagonist (antagopiR-63049) modified by phosphonothioate and cholesterol was synthesized by GenePharma Biotech Co. Ltd. (Suzhou, China) to knockdown piR-63049 *in vivo*. A total of 24 eight-week-old female Sprague Dawley rats were involved in this assay, which were then divided into the sham group, OVX treated with normal saline group, OVX treated with antagopiR control group, and OVX treated with the antagopiR-63049 group (n=6 in each group). Three days after ovariectomy, antagopiR-63049 was injected into OVX rats by tail vein injection at the dose of 10 mg/kg body weight once a week for 4 weeks. Other groups were injected with the same dose of normal saline or antagopiR control. All the rats were injected intraperitoneally with calcein green (10mg/kg body weight) at 9 days and 2 days before euthanasia 33. The rats were euthanized one week after the last antagopiR-63049 injection. The heart, liver, spleen, lung, kidney, muscle, and fat tissue of each rat were collected to observe the potential side-effects of antagopiR-63049. The right femur of each rat was collected for micro-CT and histomorphometry analysis. The left femur of each rat was collected for osteogenic gene and protein analysis.

### Micro-CT

The right femurs of rats were fixed in 4% paraformaldehyde and then scanned by a micro-CT system (SkyScan 1276, Bruker, Belgium) with the following parameters: a voltage of 80 kVp, a current of 100 μA, an exposure time of 926 ms, and a pixel size of 20 µm, according to our previous protocols 33, 37. Three-dimensional (3D) visualization images of distal metaphysis were constructed using Nrecon software. Bone static histomorphometry analyses were carried out for trabecular bone mineral density (BMD), relative bone volume (BV/TV), trabecular number (Tb.N), and trabecular thickness (Tb.Th).

### Bone histomorphometry

After micro-CT scanning, the distal metaphysis of the right femur was dehydrated with a 20% sucrose solution and frontal sections of trabecular bone were obtained from the distal femur at a thickness of 10 µm with a Leica SM2500E microtome (Leica Microsystems). The bone dynamic histomorphometry analyses for mineral apposition rate (MAR) and bone formation rate (BFR/BS) were carried out by fluorescence microscopy (Leica image analysis system, Q500MC) and Image J image analysis software (NIH).

### Histological staining of bone tissues

The right femurs of rats were decalcified in EDTA-Decalcifying-fluid (Boster Biological Technology Co., Ltd., Wuhan, China) for 4 weeks before they were embedded in paraffin, and sectioned to a thickness of 6 μm. Then the sections were stained with hematoxylin and eosin (H&E). Panoramic view of distal femurs and enlarged images (100x) were scanned by light microscopy (Olympus BX53, Olympus, Japan).

### Immunohistochemistry (IHC) and immunofluorescence (IF) staining of bone tissues

Longitudinal sections of femurs were transferred to glass slides and allowed to dry at room temperature, and incubated with primary antibodies directed against PIWI protein (Abcam, UK; ab21869; 1:200), overnight at 4 °C. Then the sections were incubated with secondary antibodies at 37 °C for 1 h and stained with 3′-diaminobenzidine (DAB). All the slides were observed and imaged via microscopy (TS2-S-SM, Nikon, Japan).

Longitudinal sections of femurs were transferred to glass slides and allowed to dry at room temperature. The sections were blocked with 10% normal goat serum for 30 minutes at room temperature and incubated with primary antibody working solution at 4 °C overnight. Then, sections were rinsed three times with phosphate-buffered saline and incubated with cyanine (cy3)-conjugated goat anti-rabbit IgG antibody in blocking buffer for 30 minutes at room temperature. The nuclei were counterstained with DAPI. Finally, the specimens were observed and imaged using a Leica DMi8 microscope (Carl Zeiss MicroImaging GHBH; Jena, Germany).

### Statistical analysis

All the results are described as mean ± standard deviation (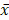
± s) and were analyzed using Prism 7.5 (GraphPad). The student's t-test was carried out to compare the difference between the two groups. Comparisons between three or more experimental groups were determined by one-way ANOVA analysis. *P* < 0.05 was regarded as the statistically significant difference. The differentially expressed piRNAs and mRNAs were those with a fold change ≥ 2 and *p* < 0.05. The correlation between piR-63049 and target mRNAs was determined by Pearson Correlation Coefficient.

## Results

### Thirteen piRNAs are dysregulated in BMSCs of OVX rats

We began the study by constructing the OVX rat and sham operation control models (Supplementary Figure 1A). BMSCs were isolated from the bone marrow of the femur and then cultured *in vitro* (Supplementary Figure 2B, C). Alizarin red staining results showed that the calcium deposition of osteoporotic BMSCs was considerably reduced after osteogenic induction (Supplementary Figure 2D, E). The expression of osteogenic marker genes and proteins (Supplementary Figure 2F, G, H) were also significantly downregulated.

**Figure 1:**
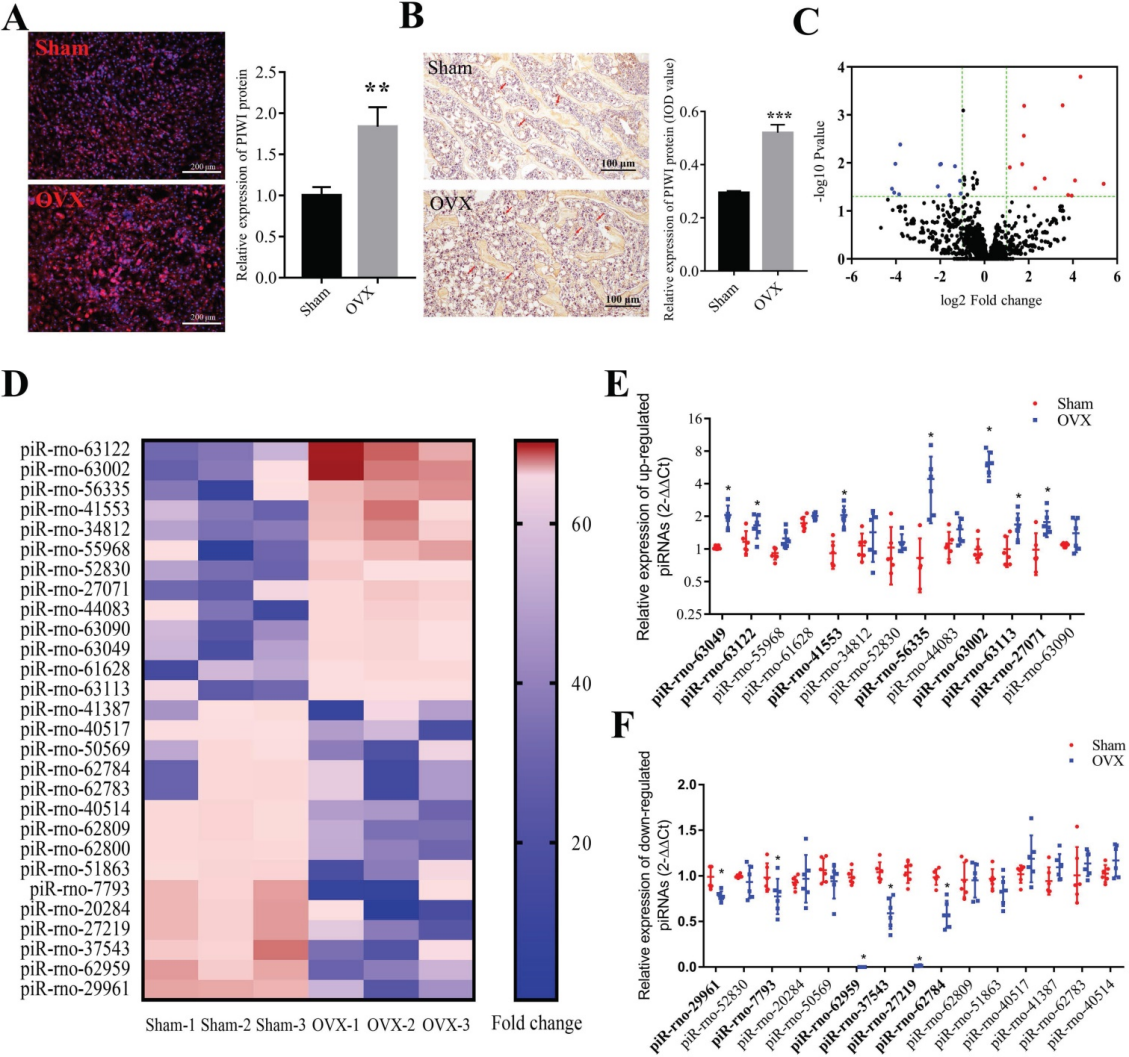
**The expression profile of piRNAs in BMSCs of OVX rats. (A)** Immunofluorescence staining and quantitative analysis show the expression level of PIWI protein in osteoporotic BMSCs (OVX) was significantly increased compared to the sham group (n=6). **(B)** The results of IHC staining for Piwi protein showed that the expression of Piwi protein significantly increased in bone tissues of OVX rats, compared to the sham group (n=6). **(C)** Volcano plot showing the fold change and P-value of each piRNA in BMSCs of OVX, compared to the Sham group. The red and blue dots represent significantly up- and down-regulated piRNAs, respectively. **(D)** Heat map showed the expression level of dysregulated piRNAs in OVX and sham rats. A total of 7 up- **(E)** and 6 down-regulated **(F)** piRNAs were validated by qPCR analysis in BMSCs of OVX and Sham rats (n=6). * *p* < 0.05, ** *p* < 0.01, *** *p* < 0.001.

Immunofluorescence staining results showed that Piwi-protein was mainly enriched in the cytoplasm, and a significant increase of Piwi-protein levels was observed in BMSCs from the OVX group compared to the sham group (Figure 1A). Consistently, IHC staining for Piwi also showed that the expression of Piwi protein significantly decreased in bone tissues of OVX rats, compared to the sham group (Figure 1B). To explore whether piRNAs were dysregulated in osteoporotic BMSCs, we performed RNA sequencing to detect the expression profile of piRNAs in osteoporotic BMSCs comparing to the sham group (Figure 1C, D). This analysis uncovered 13 up- and 15 down-regulated piRNAs whose abundance changed at least 2-fold and with a p-value < 0.05. The results were further validated in another set of OVX and sham BMSC samples (n = 6) by qPCR, which found that 7 up- and 6 down-regulated piRNAs were altered consistently with the sequencing results (Figure 1F, G).

### piR-63049 is significantly down-regulated in BMSCs after osteogenic induction while up-regulated in postmenopausal osteoporosis

**Figure 2:**
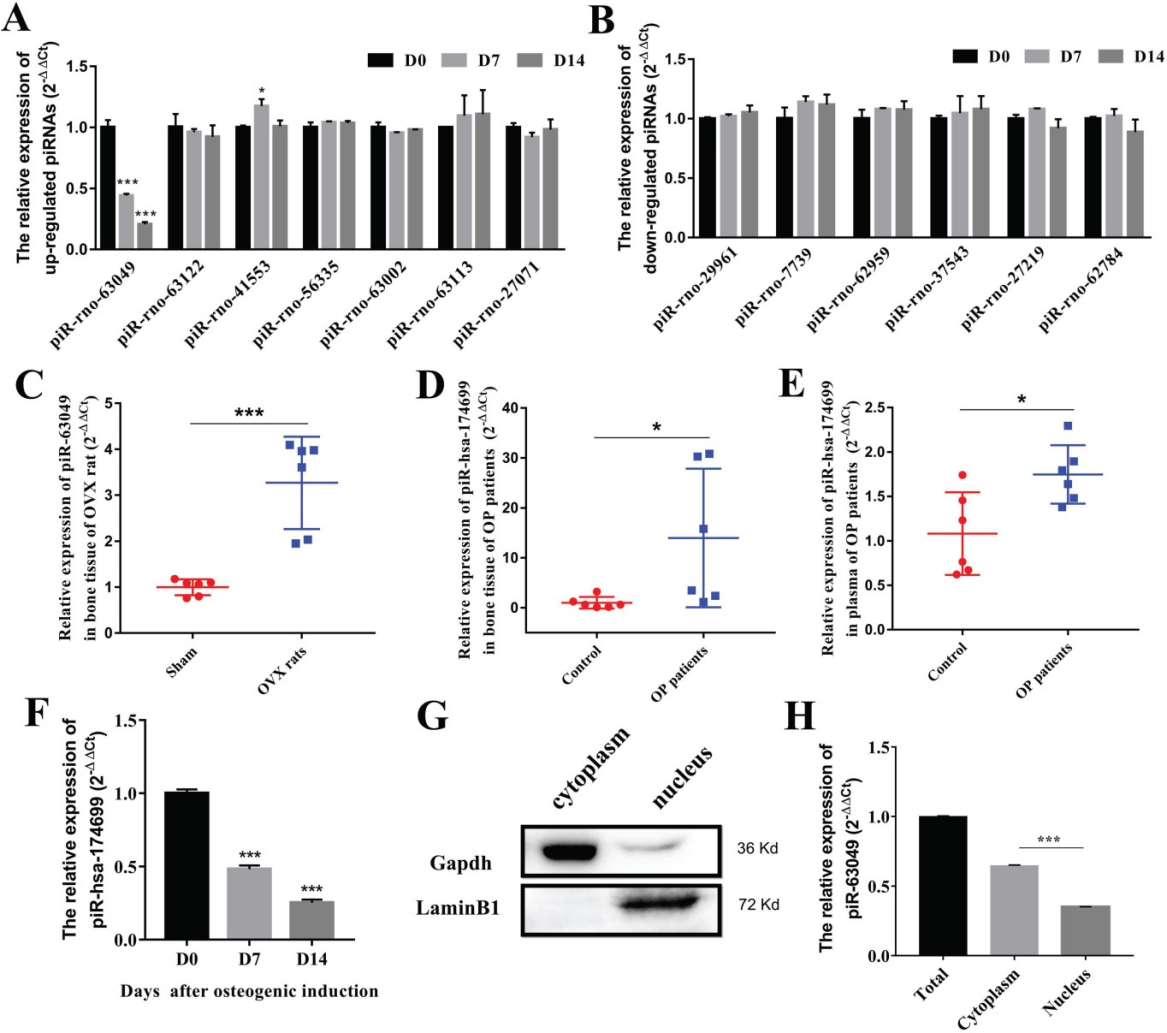
**PiR-63049 is significantly positively correlated with bone loss of osteoporosis. (A)** The expression trend of 7 up-regulated piRNAs in the osteogenesis process of BMSCs (n=3). **(B)** The expression trend of 6 down-regulated piRNAs in the osteogenesis process of BMSCs (n=3). **(C)** The expression of piR-63049 was increased dramatically in the bone tissue of OVX rats compared to that of sham controls (n=6). The expression of piR-63049 was significantly increased in the bone tissue **(D)** and plasma **(E)** of patients with osteoporosis (n=6). **(F)** The expression of piR-has-174699 (the homologous piRNA of piR-63049) was significantly decreased in the osteogenesis process of human BMSCs (n=3). **(G)** The cytoplasm and nuclei of BMSCs were separated using a nucleocytoplasmic separation kit, which was validated by the marker proteins Gapdh and Lamin. **(H)** The expression of piR-63049 was significantly higher in the cytoplasm than in the nucleus (n=3). * *p* < 0.05, *** *p* < 0.001.

To further explore whether piRNAs are involved in the osteogenic process of BMSCs, we detected the expression levels of 13 dysregulated piRNAs in BMSCs after osteogenic induction. The results indicated that only piR-63049 showed a significant decrease trend in the osteogenic process of BMSCs (Figure 2A), whereas other piRNAs showed no remarkable changes in this process (Figure 2A, B).

Further exploration showed that the expression of piR-63049 (also known as piR-hsa-174699 for human) was also significantly up-regulated in plasma of OVX rats (Figure 2C), and in the bone tissues (Figure 2D) and plasma (Figure 2E) of postmenopausal osteoporotic patients, suggesting that the expression of piR-63049 may be associated with postmenopausal osteoporosis. We also observed a significant decrease of piR-hsa-174699 (the homologous piRNA of piR-63049) in the osteogenic process of human BMSCs (Figure 2F). To confirm the intracellular localization of piR-63049, we separated RNAs from the cytoplasm and nucleus by using a nucleocytoplasmic separation Kit (Figure 2G). The results showed that the expression of piR-63049 in the cytoplasm was significantly higher than that in the nucleus of BMSCs, indicating that it was mainly localized and functioned in the cytoplasm (Figure 2H).

### *Wnt2b* is the downstream target of piR-63049

**Figure 3:**
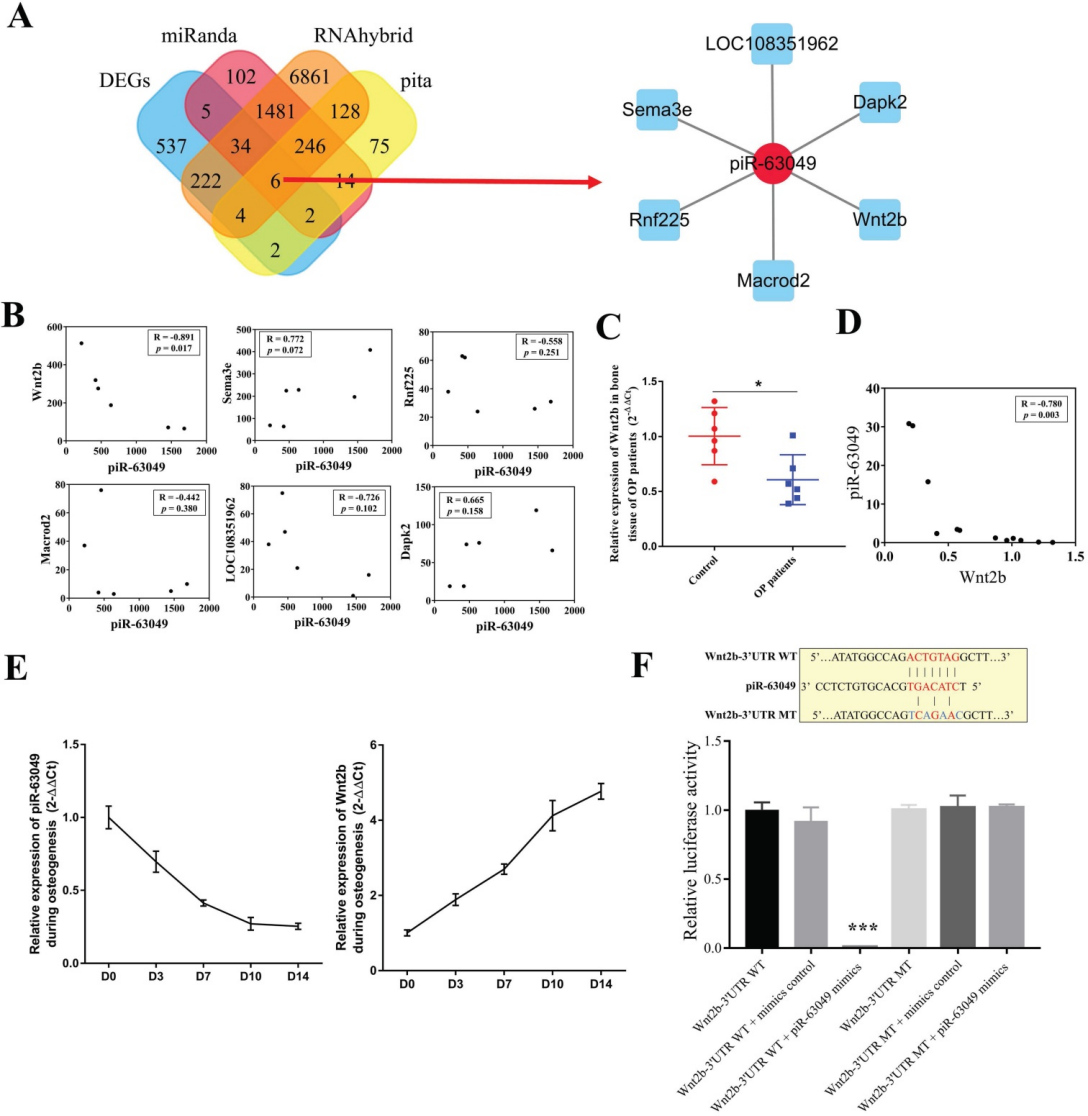
***Wnt2b* is the downstream target of piR-63049. (A)** Venn analysis showed 6 mRNAs were the potential targets of piR-63049, including *Wnt2b*, *Rnf225*, *Macrod2*, *Sema3e*, *Dapk2*, and *LOC108351962*. The regulatory network of piR-63049 and six potential target mRNAs was generated. **(B)** The gene expression correlation between piR-63049 and *Wnt2b, Rnf225*, *Macrod2*, *Sema3e*, *Dapk2*, *LOC108351962* was analyzed by Pearson correlative analysis. The results showed only Wnt2b was negatively co-related to piR-63049, while others had no significant relationship to piR-63049 (n=6). **(C)** The expression of *Wnt2b* was significantly decreased in bone tissue of patients with osteoporosis (n=6), **(D)** which was also significantly negatively correlated to the expression level of piR-63049. **(E)** The expression of piR-63049 was progressively decreased while the expression of *Wnt2b* was progressively increased in BMSCs after osteogenic induction (n=3).** (F)** The putative binding sequence and mutant sequence of piR-63049 to *Wnt2b*. The relative luciferase activity assay showing that the wildtype but not the mutant 3'-UTR sequence of *Wnt2b* could be targeted by piR-63049 mimics (n=3). * *p* < 0.05, *** *p* < 0.001.

Recent studies revealed that piRNAs can bind to the target mRNAs and inhibit their expression at the posttranscriptional level in the cytoplasm 38-40. As piR-63049 and Piwi protein were mainly enriched in the cytoplasm of BMSCs, we hypothesized that piR-63049 performs its functions by eliminating mRNAs as previous studies reported. We predicted the target mRNAs of piR-63049 and chose those mRNAs which were also differentially expressed (Supplementary Figure 3A, B, C, D) in our mRNA expression profiles by combining the MiRanda, Pita, and Rnahybrid databases. The results showed that a total of 6 mRNAs, including *Wnt2b,* ring finger protein 225* (Rnf225),* mono-ADP ribosylhydrolase 2* (Macrod2),* semaphorin 3E* (Sema3e),* death associated protein kinase 2* (Dapk2),* and *LOC108351962*, were predicted as the targets of piR-63049 (Supplementary Table 3, Figure 3A). However, only the gene expression of *Wnt2b*, which was significantly down-regulated (fold change - 2.90) in BMSCs of OVX rats, had a negative correlation with piR-63049 (R=-0.891, *p*=0.017) (Figure 3B). Further study showed that the expression of *Wnt2b* was significantly decreased in bone tissues of postmenopausal osteoporotic patients (Figure 3C), which was also negatively related to the expression level of piR-63049 (Figure 3D). Furthermore, the expression of piR-63049 was progressively decreased while the expression of *Wnt2b* was progressively increased in BMSCs after osteogenic induction, indicating that piR-63049 was negatively correlated with *Wnt2b* expression and osteogenic differentiation of BMSCs (Figure 3E). We also detected the expression of other Wnt family members after piR-63049 overexpressing or knocking-down. The results showed only the expression of Wnt2b was negatively correlated to piR-63049 (Supplementary Figure 4).

A 7-nucleotide long putative binding site of piR-63049 with the 3'UTR of *Wnt2b* was uncovered (Figure 3F), further suggesting that piR-63049 may directly regulate *Wnt2b*. Using a luciferase reporter gene plasmid containing the 3'-UTR sequence of *Wnt2b* transfected into 293T cells, we observed that piR-63049 mimics significantly decreased the luciferase activity (Figure 3F). However, no significant changes in luciferase activity were observed in 293T cells co-transfected with a variant 3'-UTR sequence of *Wnt2b* and piR-63049 mimics, suggesting that piR-63049 can directly bind to the 3 '- UTR of *Wnt2b* to regulate its expression.

### *Wnt2b* is positively associated with osteoblastic differentiation of BMSCs

**Figure 4:**
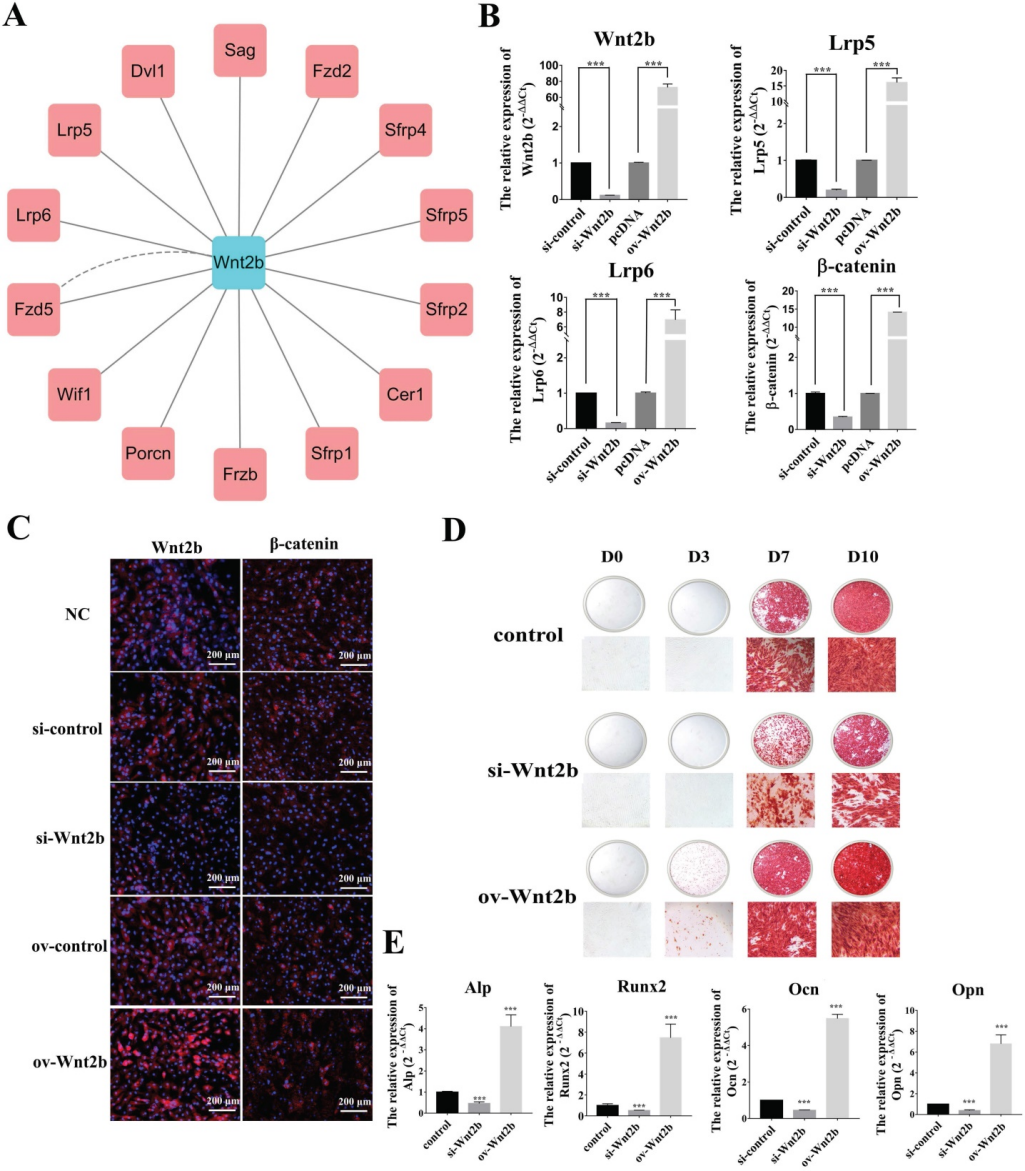
***Wnt2b* is a positive regulator for osteogenesis. (A)** The protein-protein interaction network of *Wnt2b*. **(B)** The expression of Wnt2b and Wnt/β-catenin pathway-related genes *Lrp5*, *Lrp6*, and *β-catenin* was significantly decreased upon Wnt2b knockdown but increased by Wnt2b overexpression (n=3). **(C)** Immunofluorescence analysis shows that the protein levels of *Wnt2b* and β-catenin were decreased by Wnt2b knockdown but increased by overexpression of *Wnt2b*. **(D)** Alizarin red staining shows that calcium deposition by BMSCs was decreased by knocking down *Wnt2b* but increased by overexpressing *Wnt2b*. **(E)** Expression of the osteogenesis marker genes *Alp*, *Runx2*, *Opn*, and *Ocn* was significantly decreased in BMSCs upon Wnt2b knockdown but was increased by *Wnt2b* overexpression (n=3). *** *p* < 0.001.

To explore the roles of the piR-63049-*Wnt2b* axis in bone metabolism, we first investigated the role of *Wnt2b* in the osteogenic differentiation of BMSCs. The protein-protein interaction network of *Wnt2b* was constructed by GeneMANIA using functional association data 41. A total of 14 proteins were predicted to be associated with *Wnt2b*, including low-density lipoprotein receptor-related protein 5 (*Lrp5*) and 6 (*Lrp6*), which are crucial receptors in the Wnt/β-catenin pathway (Figure 4A) 42. Therefore, we hypothesized that *Wnt2b* could regulate the Wnt/β-catenin pathway by recognizing and binding *Lrp5/6* to mediate the osteogenic differentiation of BMSCs.

We next verified the regulatory effect of *Wnt2b* on the Wnt/β-catenin pathway via knockdown and over-expression of *Wnt2b* in BMSCs. Expression of the Wnt/β-catenin pathway genes *Lrp5, Lrp6, and β-catenin* was significantly decreased upon *Wnt2b* knockdown, while their expression was remarkably increased by overexpression of *Wnt2b* (Figure 4B). Immunofluorescence further showed that the protein levels of *Wnt2b* and *β-catenin* were also substantially decreased upon *Wnt2b* knockdown, whereas they were significantly increased by overexpression of *Wnt2b* (Figure 4C). We also found that knocking down *Wnt2b* could attenuate the calcium deposition by BMSCs (Figure 4D) as well as the expression of osteogenic marker genes (*Alp, Opn, Ocn, and Runx2*), while overexpression of *Wnt2b* significantly promoted calcium deposition and expression of osteogenic marker genes (Figure 4E) in BMSCs.

### piR-63049 inhibits *Wnt2b* expression thereby suppressing the activity of the Wnt/β-catenin pathway and osteogenic differentiation of BMSCs

**Figure 5:**
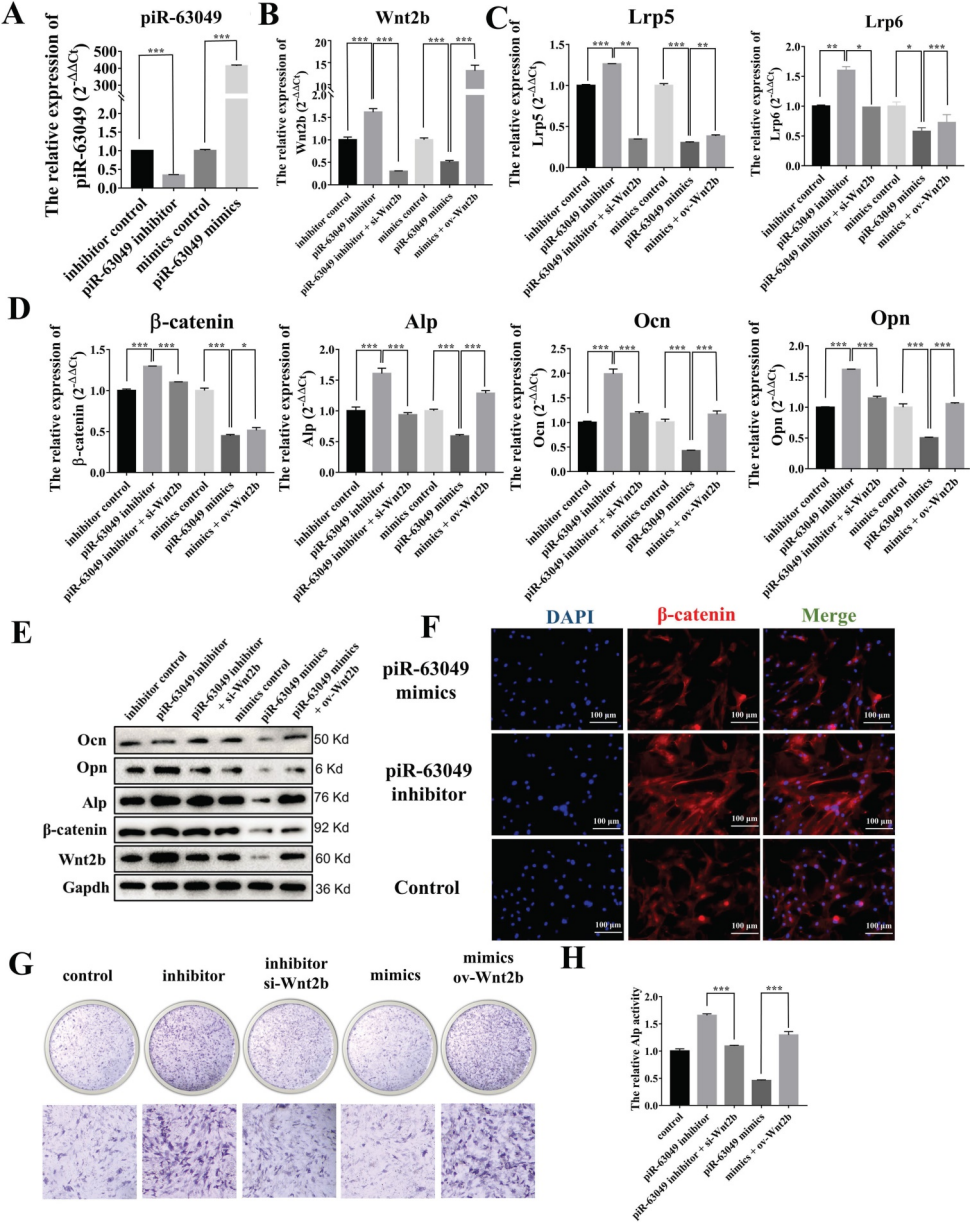
**PiR-63049 inhibits the osteogenesis of BMSCs via targeting *Wnt2b*. (A)** The expression of piR-63049 in BMSCs was significantly suppressed by transfecting piR-63049 inhibitor but increased substantially by transfection of piR-63049 mimics. (n=3). **(D)** The gene expression levels of β-catenin, Alp, Ocn, Opn are positively correlated to that of *Lrp5* and *Lrp6* (n=3). **(E)** The protein levels of Wnt2b, β-catenin, Alp, Ocn, Opn are consistent with the gene expression results. **(F)** Immunofluorescence analysis further shows that the protein level of β-catenin was significantly decreased after piR-63049 overexpression, but remarkably increased after piR-63049 knocking down. **(G, H)** Alp staining and activity examines shows that knockdown of piR-63049 can promote the osteogenesis of BMSCs, and overexpressing piR-63049 could restrain the osteogenesis of BMSCs, which could be partly reversed by co-transfecting si-Wnt2b and ov-Wnt2b respectively (n=3). * *p* < 0.05, ** *p* < 0.01, *** *p* < 0.001.

Based on our results showing that piR-63049 could bind to *Wnt2b* and that *Wnt2b* expression was positively correlated with Wnt/β-catenin pathway activity and osteogenesis, we carried out loss- and gain-of-function assays in BMSCs to further confirm whether piR-63049 could regulate osteogenic differentiation of BMSCs via targeting *Wnt2b* and downstream Wnt/β-catenin signaling. As expected, we found that knocking down piR-63049 could significantly promote *Wnt2b* gene expression while overexpressing piR-63049 could significantly inhibit *Wnt2b* gene expression (Figure 5A, B). Meanwhile, the expression of the recognition receptors (*Lrp5* and *Lrp6*) and downstream genes (*β-catenin, Ocn, Opn, Alp*) of *Wnt2b* was remarkably increased in BMSCs transfected with piR-63049 inhibitors, which could be reversed by co-transfection with *Wnt2b* siRNA (Figure 5C, D). The converse was also true, as the expression level of these genes was remarkably decreased by transfection with piR-63049 mimics, which was partially reversed by co-transfection with a *Wnt2b* overexpression plasmid (Figure 5C, D). The results of Western blot and immunofluorescence staining of β-catenin indicated that the protein expression levels of these genes were also consistent with the gene expression results, further confirming the inhibitory effect of piR-63049 on the Wnt/β-catenin pathway (Figure 5E, F). These results demonstrate that piR-63049 negatively regulates Wnt/β-catenin pathway activity by inhibiting *Wnt2b* expression.

We then explored the effect of piR-63049 on osteogenic differentiation of BMSCs via ALP staining, which showed that knocking down piR-63049 could enhance ALP activity, while overexpressing piR-63049 could attenuate ALP activity in BMSCs (Figure 5H, I). Together, these results indicate that piR-63049 can negatively regulate osteogenic differentiation of BMSCs via controlling Wnt/β-catenin pathway activity.

### Knocking-down piR-63049 *in vivo* promotes bone formation and prevents bone loss in OVX rats

Given the ability of piR-63049 to suppress osteogenic differentiation of BMSCs *in vitro*, we next sought to determine whether piR-63049 inhibition *in vivo* could prevent bone loss in ovariectomized rats via using antagopiR-63049. First, we detected the *in vitro* effects of antagopiR-63049 on the expression of Wnt2b and osteogenesis of BMSCs. The results showed that antagopiR-63049 has a significant effect on suppressing piR-63049 expression *in vitro* (Supplementary Figure 5A), thereby promoting the expression of Wnt2b (Supplementary Figure 5B) and osteogenic capability of BMSCs (Supplementary Figure 5C, D). Rats were divided into four groups: sham control rats, OVX rats treated with saline, OVX rats treated with antagopiR control, and OVX rats treated with antagopiR-63049. No significant changes in tissue weight (heart, liver, spleen, lung, and kidney) or histological features (heart, liver, spleen, lung, kidney, mussel, and fat tissue) was observed in OVX rats treated with antagopiR-63049 in comparison to the sham, saline, and antagopiR control groups (Supplementary Figure 6A, B).

**Figure 6:**
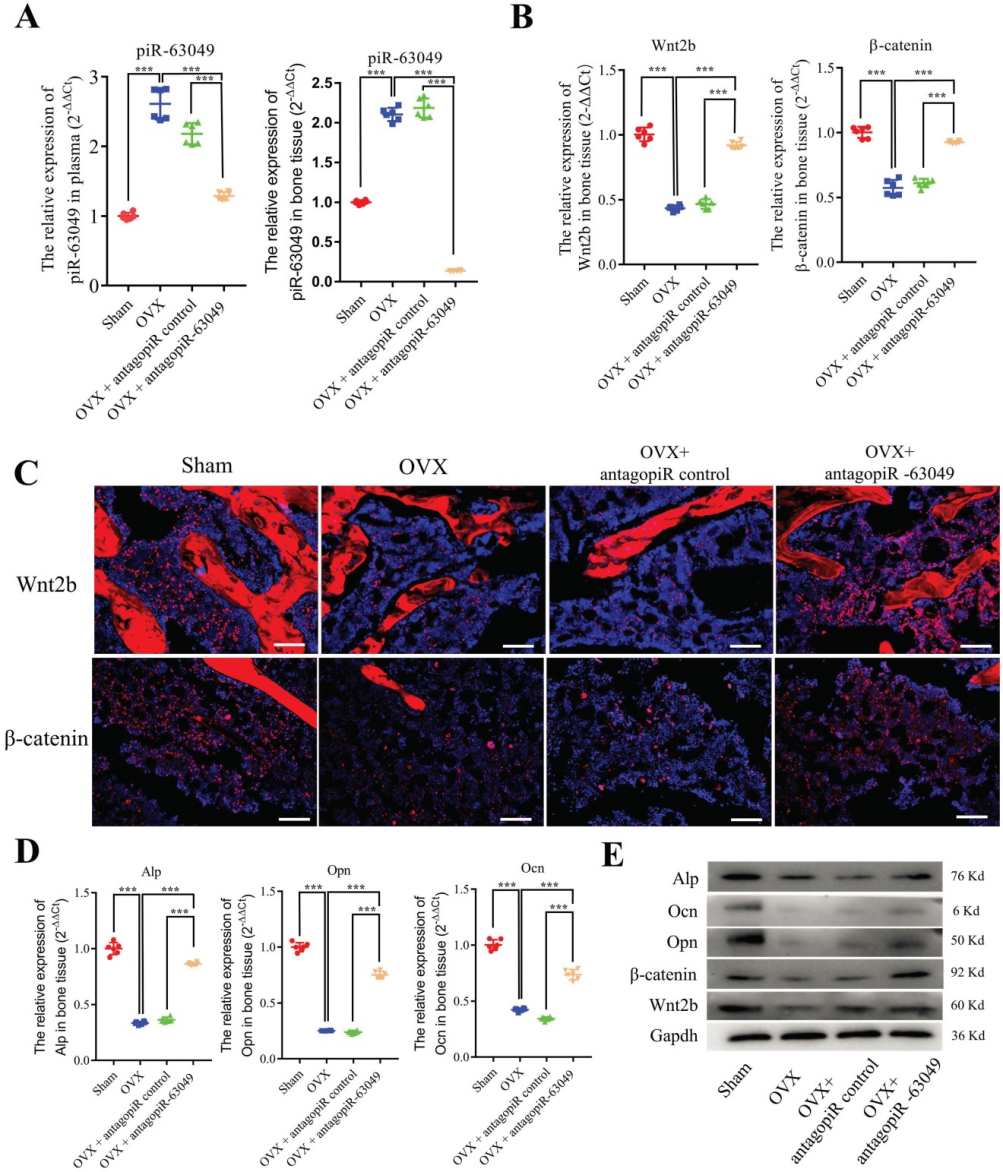
**AntagopiR-63049 treatment promotes Wnt signaling and the expression of osteogenic markers. (A)** Plasma and bone tissue levels of piR-63049 were significantly increased in OVX rats compared to sham control, but this was attenuated by antagopiR-63049 treatment (n=6). **(B)** The gene expression of *Wnt2b* and *β-catenin* in bone tissue was significantly decreased in OVX rats compared to sham control, which was attenuated in the antagopiR-63049 treatment group. **(C)** Immunofluorescence analysis shows that the protein levels of Wnt2b and β-catenin are consistent with the gene expression results. The gene **(D)** and protein **(E)** expression of osteogenic markers *Alp*, *Ocn*, *Opn* is significantly decreased in bone tissues of OVX rats compared to sham control, which was attenuated by antagopiR-63049 injection (n=6). * *p* < 0.05, ** *p* < 0.01, *** *p* < 0.001.

We next examined the expression level of piR-63049 in the bone tissue, plasma, heart, liver, spleen, lung, and kidney of each rat. The antagopiR-63049 treatment largely prevented the increase of piR-63049 in plasma and actually decreased piR-63049 levels below baseline in bone tissue (Figure 6A). However, treatment with antagopiR-63049 had no significant impact on the expression of piR-63049 in the heart, liver, spleen, lung, and kidney (Supplementary Figure 7). Moreover, the gene and protein expression levels of *Wnt2b* and *β-catenin* were significantly decreased in bone tissue of OVX rats but could be largely rescued by antagopiR-63049 treatment (Figure 6B, C). Meanwhile, the expression levels of the osteogenic marker genes *Alp, Opn,* and *Ocn* were also partially prevented by antagopiR-63049 treatment (Figure 6D, E).

**Figure 7:**
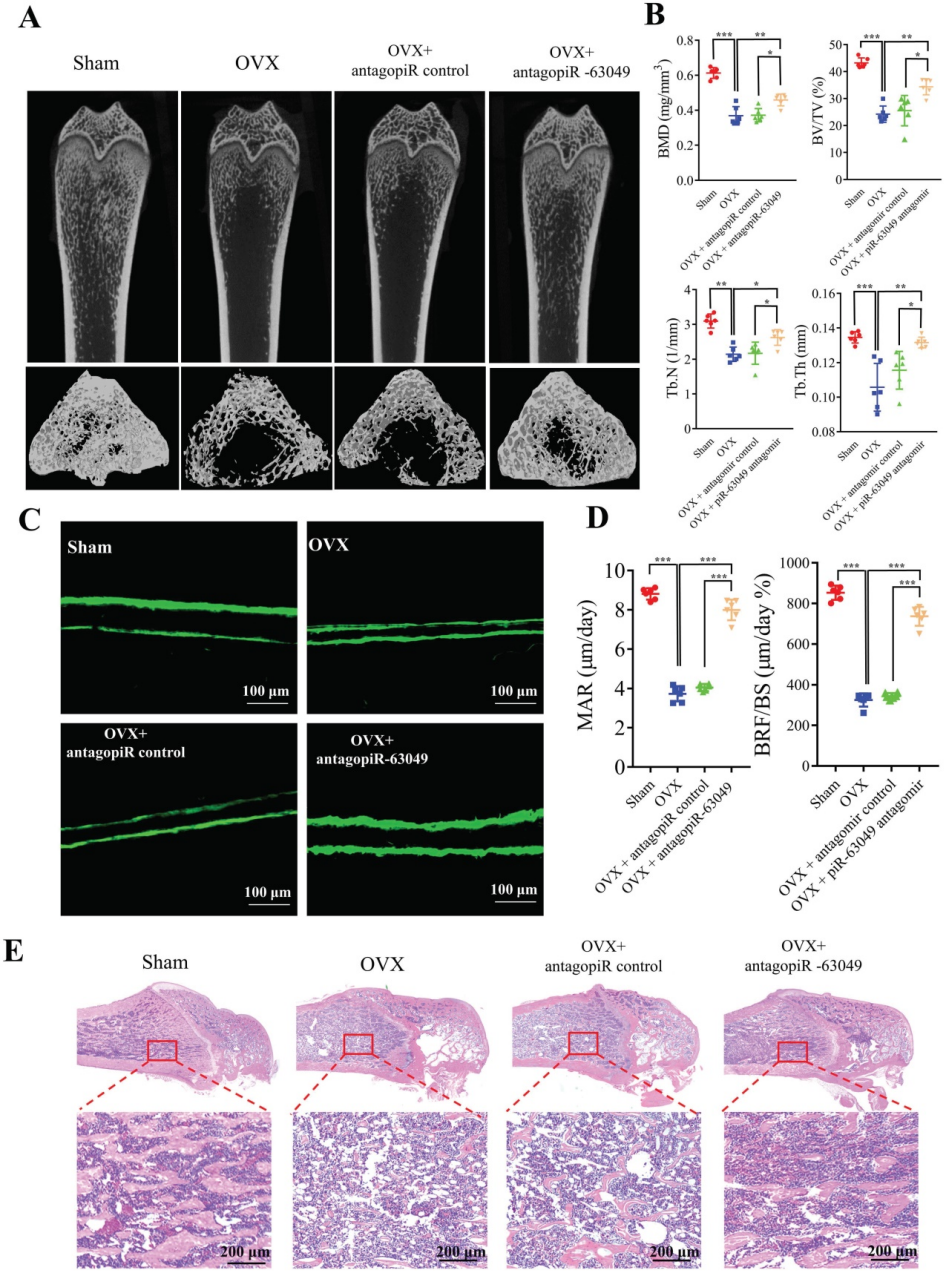
**AntagopiR-63049 treatment could prevent bone loss by promoting bone formation. (A)** Micro-CT analysis shows the bone mass in OVX rats was significantly lower than that in the sham group. However, treatment with antagopiR-63049 could significantly prevent this bone loss (n=6). **(B)** The bone phenotype parameters BMD, BV/TV, Tb.Th, and Tb.N, were remarkably decreased in OVX rats compared to the sham control group, which was significantly prevented in OVX rats treated with antagopiR-63049 (n=6). **(C)** The undecalcified bone histology showed that the width of double calcein labeling at distal femurs in the OVX group was smaller than that in the sham group, but this could be largely reversed by treatment with antagopiR-63049 (n=6). **(D)** The mineral apposition rate (MAR) and bone formation rate (BFR/BS) were significantly lower in the OVX rats compared to the sham group but were significantly higher in the antagopiR-63049 treatment group compared to that of antagopiR control (n=6). (**E**) H&E staining of bone tissues show that the cortical thickness, trabecular bone area, the number and density of osteoblasts are also reduced in the OVX rats compared to the sham group, but remarkably improved after antagopiR-63049 injection (n=6). * *p* < 0.05, ** *p* < 0.01, *** *p* < 0.001.

Thereafter, we performed micro-CT and bone histomorphometry analysis of distal femurs, which showed that the bone loss in OVX rats was notably prevented with antagopiR-63049 treatment (Figure 7A). The static bone histomorphometry indexes including BMD, BV/TV, Tb.Th, and Tb.N were all significantly decreased in OVX rats compared to the sham control group (Figure 7B). Remarkably, the decreases in each of these bone histomorphometry parameters could be partially rescued in OVX rats treated with antagopiR-63049 (Figure 7B). Analysis of undecalcified bone histology showed that the width of double calcein labeling in distal femurs in the OVX group was significantly reduced compared to that in the sham group, and this decrease was partially prevented by treatment with antagopiR-63049 (Figure 7C). Furthermore, the large decreases in MAR and BFR/BS in OVX rats were observed to be largely prevented in OVX rats treated with antagopiR-63049 (Figure 7D). Next, H&E staining also showed that the cortical thickness, trabecular bone area, the number and density of osteoblasts were also reduced in the OVX rats, but remarkably improved after knocking down piR-63049 *in vivo* (Figure 7E).

Additionally, the effect of antagopiR-63049 on osteoclast and bone resorption was also validated by ELISA and TRAP staining. The results showed that the expression of CTX-I significantly increased in OVX rats compared to the sham control group, but could be significantly rescued by antagopiR-63049 treatment (Supplementary Figure 8A). The results of TRAP staining showed that the number and size of TRAP‐positive cells were significantly increased in OVX rats, an effect which could also be significantly rescued by antagopiR-63049 treatment (Supplementary Figure 8B). Taken together, these results show that antagopiR-63049 treatment can prevent bone loss in ovariectomized rats.

## Discussion

PiRNAs were first discovered in the nucleus of germ cells, where they play essential roles in maintaining the integrity of germline DNA 43, 44. Preliminary studies have identified piRNAs as critical factors in the pathological process of certain diseases, such as cancers 45. Recently, piRNAs were also found in exosomes derived from BMSCs (BMSC-Exo) 46 and aberrantly expressed in early osteogenic and chondrogenic differentiation of BMSCs 32. To date, the roles of piRNAs in skeletal and chondral disorders still remain unclear. In the present study, we first explored the expression profile of piRNA in BMSCs of OVX rats and screened out piR-63049 as an osteogenesis-regulating piRNA, which was correlated with bone mass as it was significantly over-expressed in bone tissues and plasma of postmenopausal osteoporotic women.

Previous studies showed that piRNA mainly exerts its miRNA-like roles by targeting mRNAs in the cytoplasm of somatic cells. Gou et al. found that piRNAs were responsible for the elimination of mRNAs in spermatids during spermiogenesis via a mechanism that resembles the action of miRNAs in somatic cells 38. This mechanism was further validated by other teams 39, 40. Thereafter, a recent study showed that piR-30188 could regulate the expression of CEBPA by targeting lncRNA OIP5-AS1 at the post-transcriptional level, thus inhibiting the proliferation of glioma cells 22. Zheng et al. also discovered an inhibitory role of piR-36712 in regulating the progression of breast cancer by targeting SEPWIP RNA in the cytoplasm 47. In our study, we confirmed that piR-63049 was primarily enriched in the cytoplasm of BMSCs, indicating piR-63049 may perform its functions in the cytoplasm. Therefore, to further explore the role of piR-63049 in osteogenesis, we predicted its potential target. Interestingly, *Wnt2b*, a candidate target mRNA of piR-63049, was found negatively correlated to piR-63049. Preliminary studies have shown that* Wnt2b* is a highly conserved member of the WNT family 48. *Wnt2b* was found dysregulated in several cancers with aberrant Wnt/β-catenin pathway activity 49-53. In our study, we identified that *Wnt2b* is positively associated with osteogenesis by activating the Wnt/β-catenin signaling pathway for the first time.

**Figure 8:**
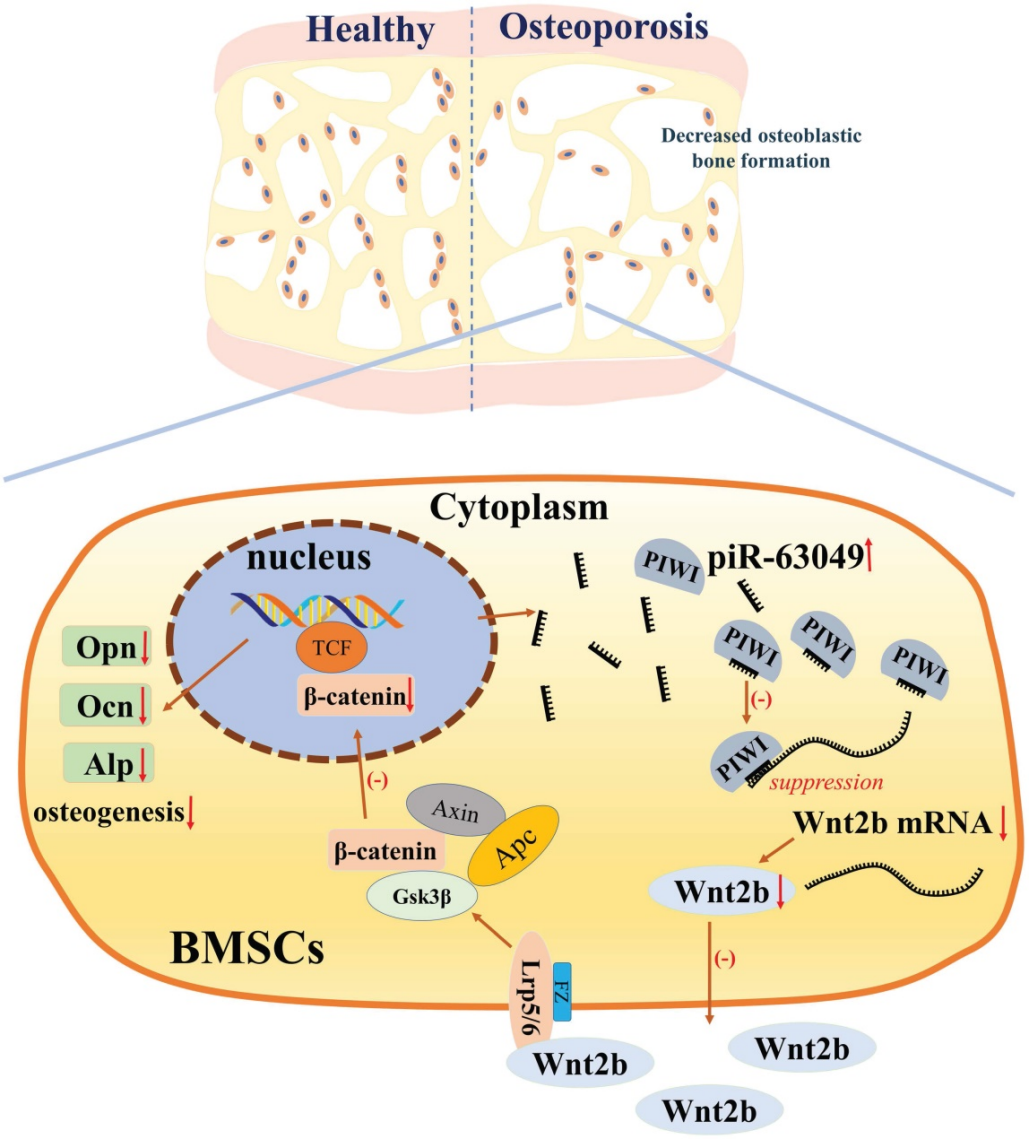
The mechanism of piR-63049 in regulating the osteogenesis of BMSCs via Wnt signaling.

The loss- and gain-of-function approaches were further performed *in vitro* to support that piR-63049 can suppress osteogenesis via targeting the Wnt2b/β-catenin pathway. Knocking down piR-63049 significantly activated the Wnt/β-catenin pathway and promoted osteogenic differentiation of BMSCs, whereas overexpressing piR-63049 suppressed the Wnt/β-catenin pathway and inhibited osteogenic differentiation of BMSCs. However, these effects of piR-63049 could be reversed by overexpressing or knocking down *Wnt2b*, respectively. These findings reveal the negatively regulatory effect of piR-63049 on osteogenesis at the molecular and cellular level (Figure 8), which may shed new light on the roles of piRNAs in regulating the process of osteogenesis and the pathogenesis of postmenopausal osteoporosis.

Disrupted bone remodeling, characterized by a decreased capacity for bone formation and increased bone resorption, is the leading cause of postmenopausal osteoporosis. Therefore, we carried out an *in vivo* assay to further address the negative role of piR-63049 in bone formation and the therapeutic effect of piR-63049 knocking down on bone remodeling. To date, the *in vivo* study of osteogenesis-related ncRNAs has been a significant challenge due to the lack of stable and specific delivery vehicles. AntagomiR is a type of specially labeled and chemically modified small molecular RNA, which can specifically inhibit the expression of microRNAs (miRNA or piRNA) 54-56. In one of our previous studies, we successfully validated the positive effect of miR-19b on promoting bone formation with the use of agomiR-19b 33. Thus, we constructed antagopiR-63049 to explore the role of piR-63049 in bone remodeling and bone metabolism *in vivo*. We found that antagopiR-63049 treatment increased the expression of osteogenic marker genes in bone tissue. Furthermore, the bone histomorphometry indexes were significantly improved in OVX rats treated with antagopiR-63049. Remarkably, the dynamic bone histomorphometry indexes were also improved by antagopiR-63049 treatment. These findings further confirmed that piR-63049 was negatively correlated with osteoporosis while inhibiting piR-63049 *in vivo* can rescue bone loss in OVX rats by promoting bone formation, which may be a useful therapeutic strategy for postmenopausal osteoporosis.

Although promising, a few limitations of the current study remain to be addressed. First, the role of piR-63049 in osteoclast-mediated bone resorption is not known and awaits further study. Additionally, although we found that antagopiR-63049 had no significant effects on the main organs analyzed in OVX rats, the antagopiR-63049 construct does not possess the ability to specifically recognize only BMSCs, and BMSC-specific delivery vehicles for ncRNAs still need to be developed and explored for targeted delivery to avoid unwanted side effects. Finally, although we found piR-63049 to be differentially expressed in the plasma of postmenopausal osteoporotic rats and patients, a larger population of human samples is still required to further verify the expression pattern of piR-63049 in postmenopausal osteoporosis and validate its utility as a biomarker.

## Conclusion

In summary, we have identified a novel ncRNA, piR-63049, which can inhibit bone formation via regulating the Wnt2b/β-catenin signaling pathway. Our study sheds new light on molecular mechanisms of osteogenesis and the pathogenesis of postmenopausal osteoporosis. Additionally, it shows that piR-63049 inhibition may be considered as a novel and viable approach for the prevention and treatment of bone loss disorders, such as postmenopausal osteoporosis.

## Supplementary Material

Supplementary figures and tables.
