# Supplementary material for: PiRNA-63049 inhibits bone formation through Wnt/β-catenin signaling pathway
Source: Int J Biol Sci. 2021 Oct 25;17(15):4409–25. doi: 10.7150/ijbs.64533 (PMC8579447; doi:10.7150/ijbs.64533)
Supplement: Supplementary file 1 — Supplementary figures and tables. [file ijbsv17p4409s1.pdf]

Supplementary Table 1 Demographics of the osteoporosis patients involved in this study

	Control	OP
Patient number	6	6
Gender	Female	Female
Age	63.75 ± 2.99	66.25 ± 1.89
Bone density (T-score ± SD)	-0.18 ± 0.55	-2.93 ± 0.10

Supplementary Table 2 Primers used in this study

Gene symbol	Accession number	Forward (3'-5')	Reverse (3'-5')
Wnt2b	NM_004185.4	GGGGCACGAGTGATCTGTG	GCATGATGTCTGGGTAACGCT
Lrp5	NM_002335.4	AACGGCAGGACGTGTAAGG	AGCGAGATCCTCCGTAGGTC
Lrp6	NM_002336.3	TTTATGCAAACAGACGGGACTT	GCCTCCAACTACAATCGTAGC
β-catenin	XM_006712983.2	CATCTACACAGTTTGATGCTGCT	GCAGTTTTGTCAGTTCAGGGA
Ocn	NM_199173.6	CACTCCTCGCCCTATTGGC	CCCTCCTGCTTGGACACAAAG
Opn	NM_001040058.2	CTCCATTGACTCGAACGACTC	CAGGTCTGCGAAACTTCTTAGAT
Alp	NM_125429.4	ACTGGGGCCTGAGATACCC	TCGTGTTGCACTGGTTAAAGC
Gapdh	NM_002046.7	TGTGTCCGTCGTGGATCTGA	TTGCTGTTGAAGTCGCAGGAG

Supplementary Table 3 The sequencing data of potential target mRNAs of piR-63049 in BMSCs of OVX rats

Gene symbol	Expression trend	Fold change	P-value
Wnt2b	down	-2.90947877	0.00315
Rnf225	down	-2.067328965	0.047589
Macrod2	down	-6.450895959	0.006066
Sema3e	up	2.347193869	0.014987
Dapk2	up	2.428486444	0.040763
LOC108351962	down	-4.430667559	0.009633

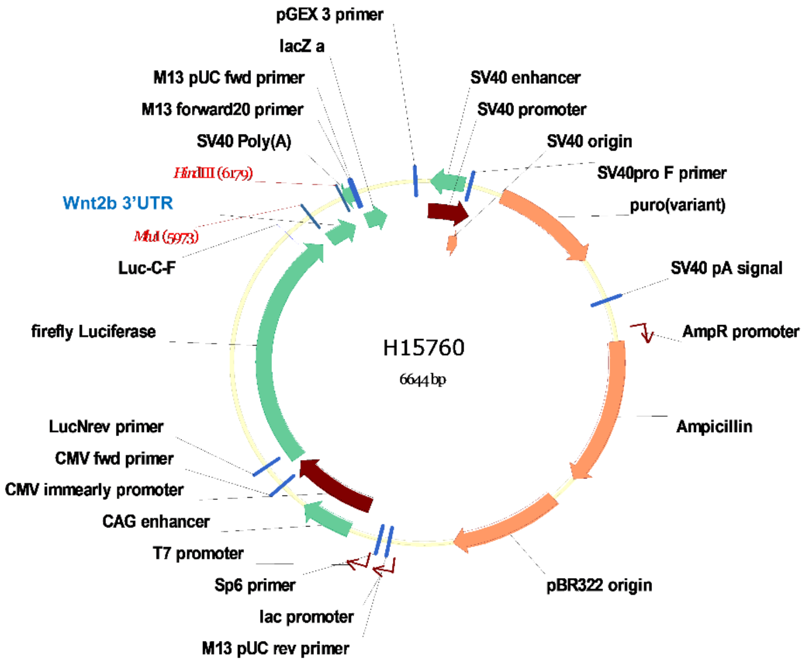

Supplementary Figure 1 The backbone of luciferase reporter gene plasmid containing Wnt 3'UTR.

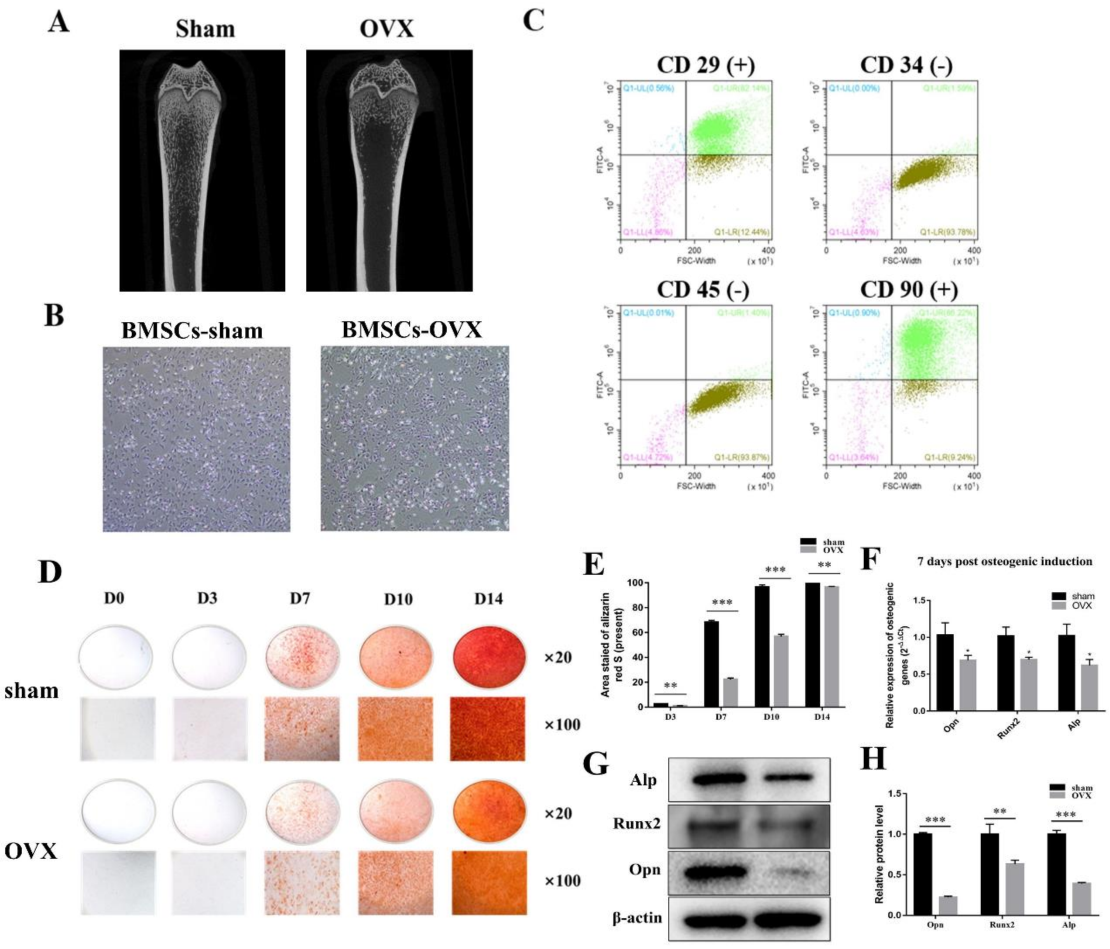

Supplementary Figure 2 The osteogenic differentiation capacity of osteoporotic BMSCs was significantly decreased. (**A**) The bone density of femurs from OVX rats was significantly decreased compared to sham control rats. (**B**) The bone marrow mesenchymal stem cells of OVX and sham control rats. (**C**) Flow cytometry showing that the isolated BMSCs were negative for surface differentiation antigens CD34 and CD45, while they were positive for CD29 and CD90. (**D**, **E**) Alizarin red staining shows that the BMSCs of OVX rats have decreased osteogenic differentiation capacity compared to those from sham control rats (n=3). (**F**) Expression of the osteogenic marker genes Opn, Runx2, and Alp was significantly reduced in BMSCs of OVX rats (n=3). (**G**, **H**) The protein levels of osteogenic markers were also significantly decreased in BMSCs of OVX rats (n=3). * $p < 0.05$, ** $p < 0.01$, *** $p < 0.001$.

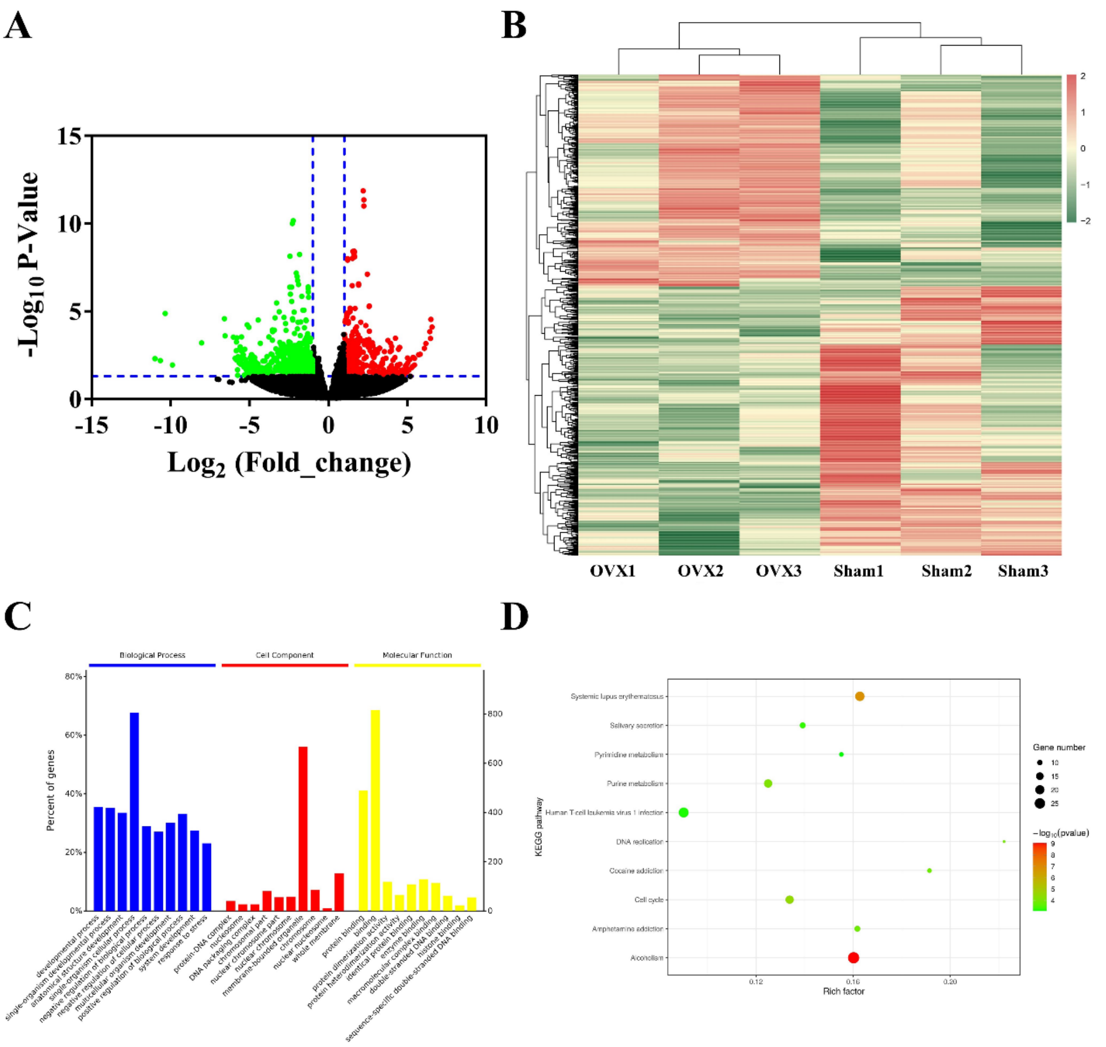

Supplementary Figure 3 The differential expression profile of mRNAs in BMSCs of OVX rats. (**A**) Volcano plot of mRNAs expressed in BMSCs of OVX rats. (**B**) Heatmap of differentially expressed mRNAs in OVX rats. (**C**) GO enrichment analysis of differentially expressed mRNAs. (**D**) KEGG pathway enrichment analysis of differentially expressed mRNAs.

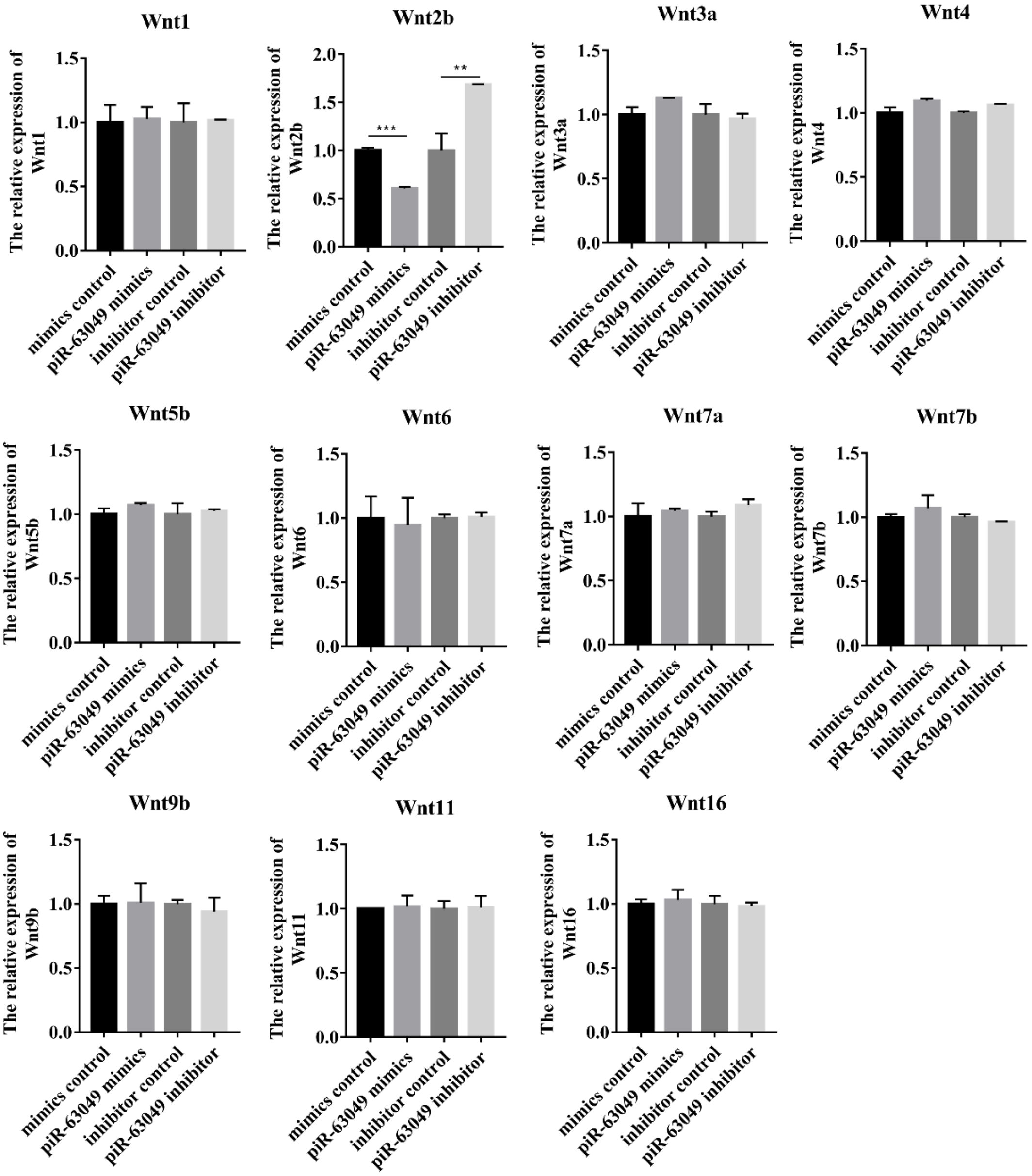

Supplementary Figure 4 The expression of Wnt1, Wnt2b, Wnt3a, Wnt4, Wnt5a, Wnt6, Wnt7a, Wnt7b, Wnt9a, Wnt11, and Wnt16 after piR-63049 overexpressing or knocking-down (n=6). ** $p < 0.01$, *** $p < 0.001$.

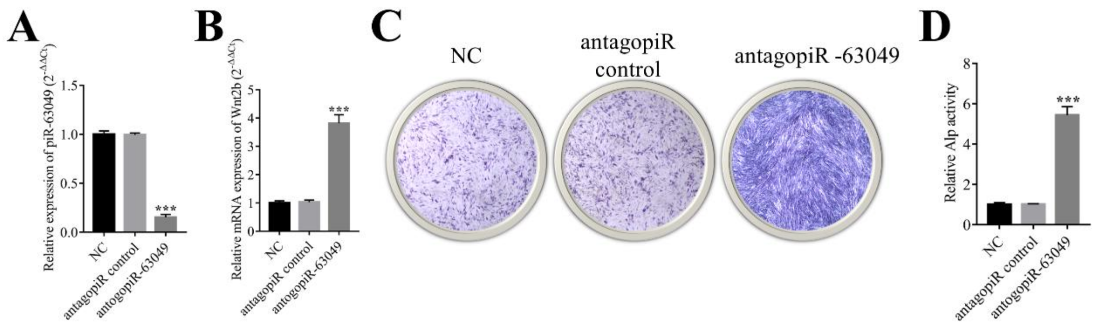

Supplementary Figure 5 *In vitro* effects of antagopiR-63049. (A) AntagopiR-63049 transfection significantly suppressed the expression of piR-63049 but promoted the expression of Wnt2b (n=3). (B) The results of ALP staining showed that antagopiR-63049 transfection could significantly promote the osteogenic capability of BMSCs (n=3). *** $p < 0.001$.

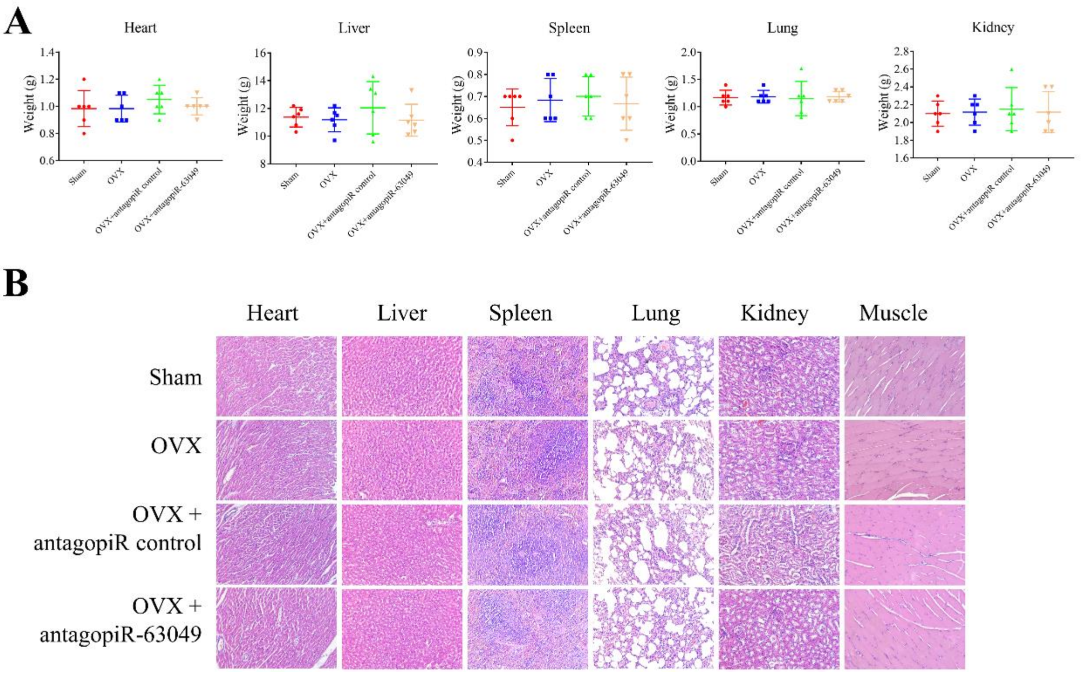

Supplementary Figure 6 Treatment with antagopiR-63049 had no significant impact on primary organs (**A**) No significant change in tissue weight of internal organs (including heart, liver, spleen, lung, and kidney) was observed in OVX rats treated with antagopiR-63049 (n=6). (**B**) No significant histological change in key tissues (including heart, liver, spleen, lung, kidney, and muscle) was observed in OVX rats treated with antagopiR-63049 (n=6).

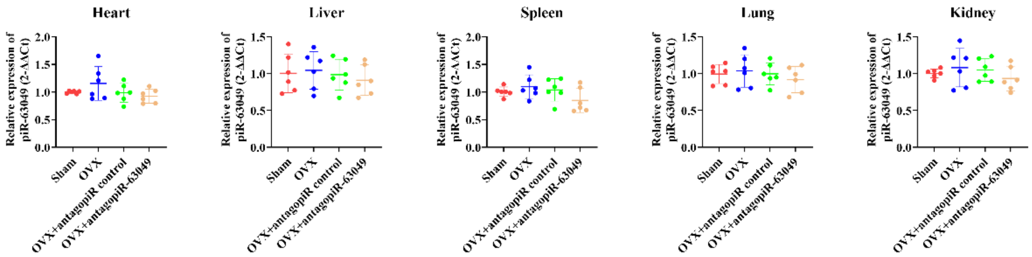

Supplementary Figure 7 Treatment with antagopiR-63049 had no significant impact on the expression of piR-63049 in heart, liver, spleen, lung and kidney (n=6).

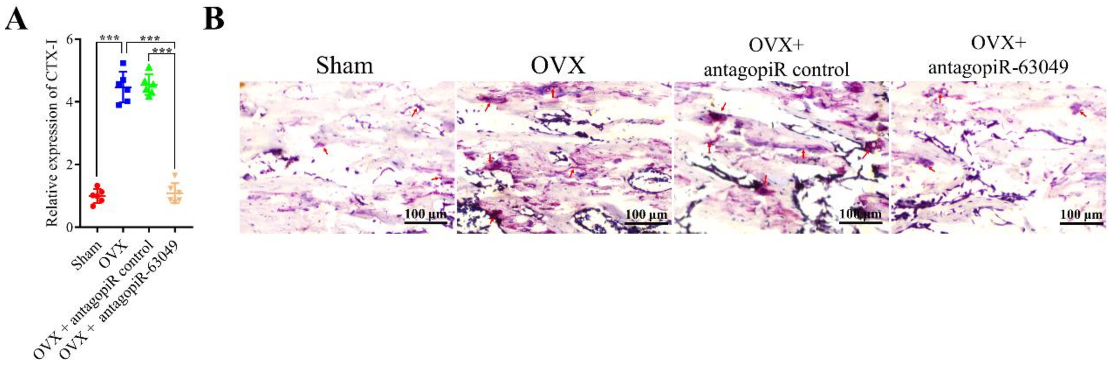

Supplementary Figure 8 AntagopiR-63049 treatment inhibits bone resorption. (A) The results of ELISA showed that the expression of CTX-I was significantly increased in OVX rats compared to the Sham group, but could be significantly rescued by antagopiR-63049 treatment (n=6). (B) The results of TRAP staining showed that the number and size of TRAP-positive cells were significantly increased in OVX rats, an effect which could be significantly rescued by antagopiR-63049 treatment (n=6). *** $p < 0.001$.
